# A wide-field and high-resolution lensless compound eye microsystem for real-time target motion perception

**DOI:** 10.1038/s41378-022-00388-w

**Published:** 2022-07-22

**Authors:** Li Zhang, Haiyang Zhan, Xinyuan Liu, Fei Xing, Zheng You

**Affiliations:** 1 Department of Precision Instrument, Tsinghua University, Beijing, 100084 China; 2 State Key Laboratory of Precision Measurement Technology and Instrument, Tsinghua University, Beijing, 100084 China; 3 Beijing Advanced Innovation Center for Integrated Circuits, Beijing, 100084 China

**Keywords:** Optical sensors, Micro-optics, Electrical and electronic engineering

## Abstract

Optical measurement systems suffer from a fundamental tradeoff between the field of view (FOV), the resolution and the update rate. A compound eye has the advantages of a wide FOV, high update rate and high sensitivity to motion, providing inspiration for breaking through the constraint and realizing high-performance optical systems. However, most existing studies on artificial compound eyes are limited by complex structure and low resolution, and they focus on imaging instead of precise measurement. Here, a high-performance lensless compound eye microsystem is developed to realize target motion perception through precise and fast orientation measurement. The microsystem splices multiple sub-FOVs formed by long-focal subeyes, images targets distributed in a panoramic range into a single multiplexing image sensor, and codes the subeye aperture array for distinguishing the targets from different sub-FOVs. A wide-field and high resolution are simultaneously realized in a simple and easy-to-manufacture microelectromechanical system (MEMS) aperture array. Moreover, based on the electronic rolling shutter technique of the image sensor, a hyperframe update rate is achieved by the precise measurement of multiple time-shifted spots of one target. The microsystem achieves an orientation measurement accuracy of 0.0023° (3σ) in the *x* direction and 0.0028° (3σ) in the *y* direction in a cone FOV of 120° with an update rate ~20 times higher than the frame rate. This study provides a promising approach for achieving optical measurements with comprehensive high performance and may have great significance in various applications, such as vision-controlled directional navigation and high-dynamic target tracking, formation and obstacle avoidance of unmanned aerial vehicles.

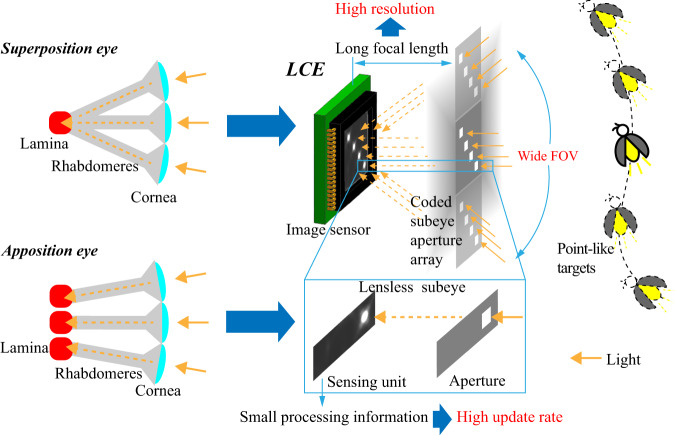

## Introduction

Realizing the compatibility of a wide field of view (FOV), high resolution and a high update rate is a challenging scientific problem in the field of optical measurement. The orientation measurement of moving targets with high resolution and a high update rate in a wide FOV is also a common requirement in various applications, such as vision-controlled directional navigation and high-dynamic target tracking, formation and obstacle avoidance of unmanned aerial vehicles (refs. ^1–6^). Optical systems in nature can be roughly divided into three types: human eyes, fish eyes and compound eyes (ref. ^7^). The distinctive characteristics of these architectures can provide inspiration to meet the increasing requirements for high-performance optical measurement. Unlike vertebrate single-aperture eyes, compound eyes (refs. ^8,9^) are multiaperture systems made up of small eyes with different viewing angles; thus, their FOV can be as wide as that of fish eyes. Moreover, due to independent sensing neurons, with each corresponding to an ommatidium, and the parallel processing procedure, compound eyes have the advantages of a high update rate and high sensitivity to motion that human eyes and fish eyes do not have. If the high resolution of human eyes can be achieved in compound eyes without largely complicating the structure, high-performance optical measurement systems can be realized.

There are diverse kinds of artificial compound eyes, of which the most common one is composed of a planar microlens array (or a lens-free structure) and a planar pixel array (refs. ^10–22^). As a result, the FOV is limited by the planar-distributed subeyes. To achieve a wide FOV, artificial compound eye systems with both curved lens arrays and photosensitive arrays are designed from the bionic perspective (refs. ^23–25^). Relying on flexible electronic techniques, curved photosensitive arrays are difficult to manufacture and incompatible with existing planar image sensors. Therefore, some studies have turn to a compound eye system combining a curved lens array with a planar pixel array. Due to the mismatch of the two components, it is necessary to design a relay device (refs. ^26–31^) or waveguide device (refs. ^32,33^). However, these devices only serve the purpose of light transmission and do not increase the focal length, retaining the low resolution typical for common compound eye systems. In addition to the low resolution and manufacturing difficulty (ref. ^34^), most existing compound eye systems focus on the restoration of observed images, and there are a few studies (refs. ^24,26,30–32^) on precise target motion measurement (See Supplementary Table 1 for a comparison of the performance of different compound eye systems.).

**Fig. 1:**
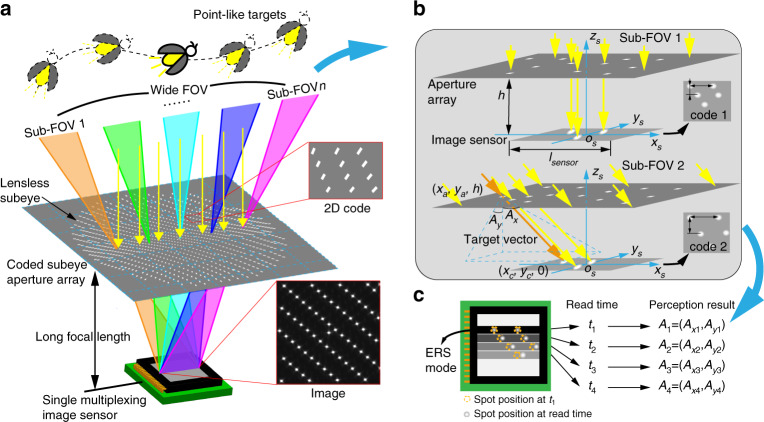
Principle of LCE. **a** Schematic diagram of the LCE with a wide FOV and long focal length. **b** Principle of FOV recognition using a coded aperture array. **c** Hyperframe update rate realization based on the ERS imaging technique

Here, we develop a lensless compound eye (LCE) microsystem with a wide FOV, high resolution and high update rate. As shown in Fig. 1a, the microsystem consists of a planar-coded subeye aperture array and a planar multiplexing image sensor. Different from existing ultrathin planar artificial compound eye systems, LCE has a long focal length to fuse the high-resolution advantage of single-aperture cameras. To overcome the challenge of FOV decline caused by the long focal length, the microsystem splices multiple sub-FOVs, images targets distributed in a panoramic range into a single multiplexing image sensor, and codes the subeye aperture array for distinguishing the targets from different sub-FOVs (Fig. 1b). A wide-field and high resolution are simultaneously realized in a simple and easy-to-manufacture microelectromechanical system (MEMS) aperture array. Moreover, based on the electronic roller shutter (ERS) imaging technique of the image sensor, precise measurement of multiple time-shifted spots of one target is performed to achieve a hyperframe update rate (Fig. 1c). The LCE is a novel artificial compound eye that realizes real-time motion perception by precise and fast orientation measurement for point-like targets. The study provides a promising approach for achieving optical measurements with comprehensive high performance, even though the capacities are restricted with each other. It has great significance in various applications, such as vision-controlled directional navigation and high-dynamic target tracking, formation and obstacle avoidance of unmanned aerial vehicles (refs. ^1–6^).

## Results

### Principle of high-performance LCE

The LCE realizes target motion perception through precise and fast orientation measurement. The perceived target of the LCE is a point-like target whose opening angle relative to aperture is much smaller than the sub-FOV of the LCE. When a point-like target is in the FOV of the LCE, the emitted or reflected light passes through the coded subeye aperture array and forms a series of projected coded spots on the single multiplexing image sensor. By decoding the position information of the spots, we can match the spots with the corresponding subeye apertures using perceptual algorithms (see Supplementary Note 1). Then, the orientation of the perceived target in the LCE coordinate system (*o*_*s*_*x*_*s*_*y*_*s*_*z*_*s*_) can be determined. Specifically, in the LCE coordinate system with the center of the image sensor as the origin, the target orientation can be represented by the angle vector (*A*_*x*_, *A*_*y*_) formed by the *z*_*s*_ axis and the projection of the incident light on the *o*_*s*_*x*_*s*_*z*_*s*_ and *o*_*s*_*y*_*s*_*z*_*s*_ planes (Fig. 1b) if the light can be assumed to be near parallel (see Supplementary Note 2 for cases of nonparallel light). The target orientation vector (*A*_*x*_, *A*_*y*_) that we measure can be calculated by

1 $$A_x = \arctan \left( {\frac{{x_c - x_a}}{h}} \right)$$

2 $$A_y = \arctan \left( {\frac{{y_c - y_a}}{h}} \right)$$

where *h* is the distance from the aperture array to the image sensor, (*x*_*a*_, *y*_*a*_, *h*) is the coordinate of a matched subaperture center, and (*x*_*c*_, *y*_*c*_, 0) is the coordinate of the corresponding spot.

In the measurement, there exists a fundamental tradeoff between high resolution and a wide FOV. For single-aperture optical systems, a long focal length results in high resolution but a narrow FOV. Existing studies have achieved a large FOV by using multiple high-resolution single-aperture systems as a multiaperture system, which leads to a large instrument structure, data volume surge and low resource utilization (refs. ^35,36^). In this paper, multiple sub-FOV splicing and single-image sensor multiplexing are adopted to solve the contradiction between a large FOV and high resolution in a simple and easy-to-manufacture MEMS aperture array. The lens array size of a common planar artificial compound eye is close to the size of the image sensor, and the lens is near the image sensor (refs. ^10–12,16,20^). To achieve high resolution, we lift the planar subeye aperture array away from the image sensor to create a long focal length. At the same time, the size of the subeye aperture array is much larger than that of the image sensor, so the incident light from a wide range can reach the image sensor through different regions of the subeye aperture array, which ensures the realization of a wide FOV. The subeye aperture array is coded to recognize which region of the array or which sub-FOV the light is incident from.

Furthermore, we utilize the ERS imaging mode of the image sensor to enable the capability of tracking high-dynamic targets. The subeyes are densely arranged to ensure that photons from a target pass through multiple subeyes and form corresponding spots. The spots located on different rows of the image sensor are exposed at different moments (Fig. 1c) due to the ERS technique and are separately measured using a 1D morphology approach (ref. ^37^). By acquiring and processing the images line by line, instead of frame by frame, we realize a hyperframe update rate in the LCE for real-time orientation measurement.

### LCE design and optimization

The basic optical parameters of the LCE are first determined for the convenience of subsequent analysis. Considering that several instruments can be used together to achieve a FOV of 360°, we choose 120° as the FOV of the LCE. Since the existing spot-centering methods can reach subpixel (submicron) accuracy, we set 7 mm as the focal length of the LCE to ensure arc-second angular resolution (see LCE instrument and performance analysis) without greatly increasing the volume of the microsystem. Next, we mainly optimize the subeye aperture array, the key component of the LCE, from two aspects: the aperture size for image quality and the aperture distribution for sub-FOV recognition.

**Fig. 2:**
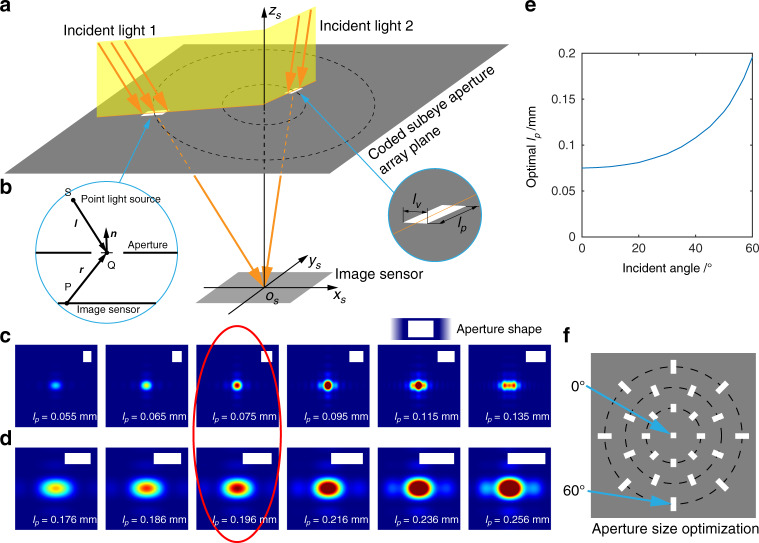
Optimization of aperture size. **a** Schematic diagram of the radially arranged apertures around the *z*_*s*_ axis. **b** Parameters used in diffraction analysis. **c** Simulated profiles for vertically incident light over different *l*_*p*_. **d** Simulated profiles for the 60° incident light over different *l*_*p*_. **e** Optimal *l*_*p*_ over incident angle. **f** Schematic diagram of the aperture size optimization result

The aperture size affects the spot profile and then affects the measurement resolution and update rate of the microsystem. Here, different from a circular aperture with only one constraint parameter, we choose a rectangle with two orthogonal constraint parameters as the aperture shape. For each aperture, we optimize the two parameters by maximizing the energy concentration of the spot profile formed by the incident light from the aperture to the image sensor center based on Fresnel–Kirchhoff diffraction (see Materials and methods for details). For convenience, we radially place the apertures around the *z*_*s*_ axis (Fig. 2a) so that the incident light we analyze only has a projection component on one side (with the length of *l*_*p*_) of each rectangular aperture and has no components on the other side (with the length of *l*_*v*_). Then, the optimal *l*_*p*_ is different for each incident angle, while the optimal *l*_*v*_ remains unchanged. Note that each incident angle corresponds to a concentric circle in the subeye aperture array plane. The apertures at one concentric circle have the same *l*_*p*_ but different rotations, and all the apertures have the same *l*_*v*,_ which equals the *l*_*p*_ of the aperture on the *z*_*s*_ axis (Fig. 2f). The simulated profiles for the vertically incident light over different *l*_*p*_ are shown in Fig. 2c, and those for the 60° incident light are shown in Fig. 2d (see Supplementary Note 3). Finally, *l*_*v*_ is optimized to be ~0.075 mm, and *l*_*p*_ is optimized to vary over the incident angle, as shown in Fig. 2e (for the specific value, see Supplementary Table 3).

**Fig. 3:**
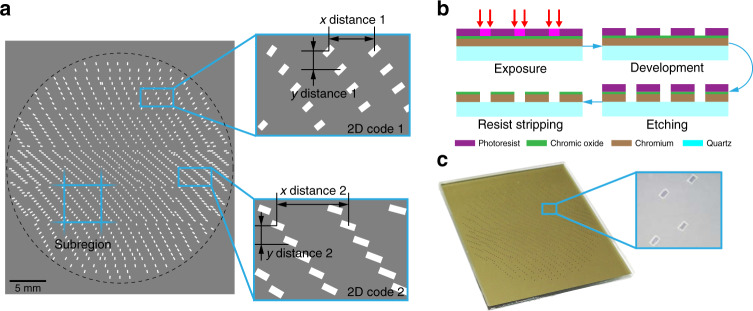
Design of the aperture distribution. **a** Schematic diagram of the coded subeye aperture array. Each subregion has a unique type of 2D code. **b** Manufacturing process of the subeye aperture array. **c** Picture of the subeye aperture array and its appearance from an optical microscope

The aperture distribution is designed and coded to distinguish which sub-FOV or aperture a spot on the multiplexed image sensor is from. To achieve a wide FOV of 120° at a focal length of 7 mm, the subeye apertures are distributed in a circular area with a diameter of ~30 mm. We then divide the subeye aperture array into multiple subregions, each of which has the same size as the image sensor. As shown in Fig. 3a, we make the distance between adjacent apertures different in different subregions to realize 2-dimensional (2D) coding. Some distance values with obvious differences are used as coding values (such as 1.3 mm, 1.4 mm, and 1.5 mm in the *x* and *y* directions). The acquired images contain the coding information of one or more subregions. By analyzing the positions of the spots, the subregions and apertures corresponding to the spots can be determined by the perception algorithm (see Supplementary Note 1). The subeye aperture array is finally designed to have 1026 lensless apertures located in 37 subregions (Fig. 3c**)**, and its manufacturing process is easy (Fig. 3b and Materials and methods).

### LCE instrument and performance analysis

**Fig. 4:**
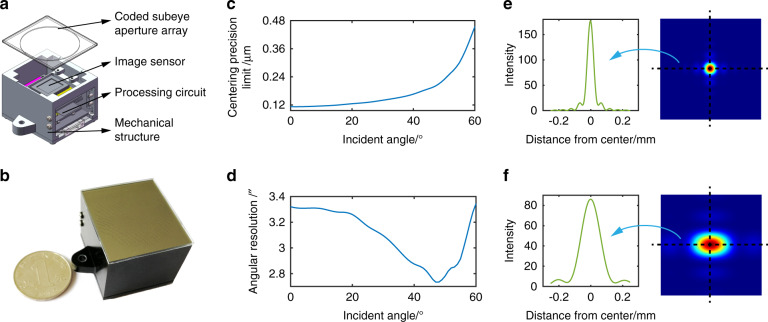
LCE instrument and performance analysis. **a** Structure of LCE. **b** Picture of the LCE instrument. **c** Centering precision limit over incident angle. **d** Angular resolution over incident angle. **e** Spot profile and intensity distribution corresponding to the vertically incident light. **f** Spot profile and intensity distribution corresponding to the 60° incident light

The structure and instrument picture of the LCE are shown in Fig. 4a, b. The subeye aperture array contains 1026 apertures that are distributed in a circular area with a diameter of ~30 mm. The image sensor is placed 7 mm behind the subeye aperture array, which contains 2048 × 2048 pixels, each with a length of 2.4 μm. The size of the instrument is 32 mm × 36 mm × 28.3 mm. Its weight is 44.4 g, and its power consumption is approximately 1.1 W (see Supplementary Table 2 for more details).

The FOV of the LCE depends on the geometric arrangement of the subeye apertures and the image sensor. The resolution for orientation measurement is determined by the focal length (*h*) of the LCE and the minimum distinguishable displacement (Δ*l*) of the spot, and this Δ*l* is determined by the centering precision limit related to the spot profile (ref. ^38^). The update rate is affected by the frame rate and the number of spots that can be distributed in different exposure rows of the detector. Details of the performance analysis are illustrated in the Materials and methods. The analysis shows that the FOV of the LCE reaches 120°. The centering precision limit over the incident angle is shown in Fig. 4c, and the angular resolution is shown in Fig. 4d, which is slightly different for various incident angles, with an average of 3.1″ (0.0008°). Considering the size of the optimized spot for the vertically incident light (Fig. 4e) and that for the 60° incident light (Fig. 4f), the maximum update rate can be ~30–100 times higher than the frame rate of the image sensor depending on the incident angle (see Materials and methods) if the apertures are distributed densely. For a frame rate of 50 Hz, the maximum update rate can reach 1.5 kHz–5 kHz.

### Orientation measurement for static and dynamic targets

**Fig. 5:**
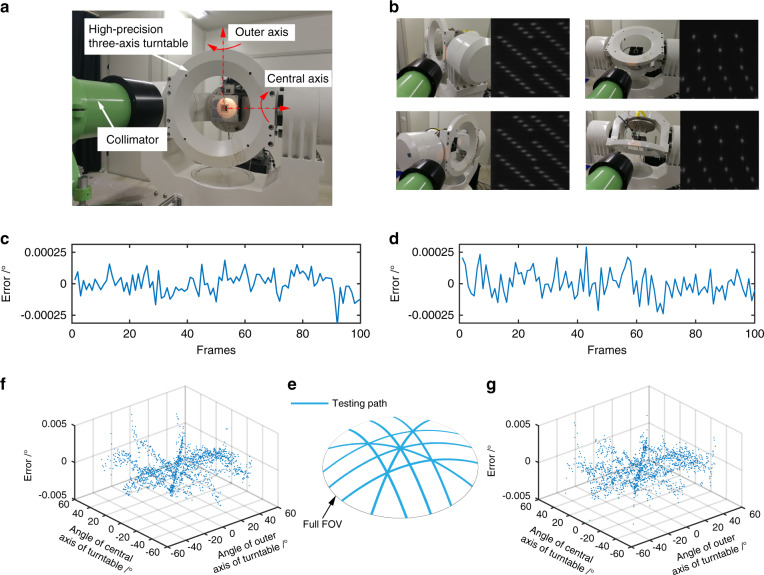
Experimental setup and results for static target orientation measurement. **a** Experimental setup. **b** Large rotations made by the turntable for wide-FOV orientation measurement verification. **c** Orientation measurement errors in the *x* direction for 100 images sampled at one position. **d** Orientation measurement errors in the *y* direction for 100 images sampled at the position. **e** Testing paths for accuracy assessment. **f** Orientation measurement errors in the *x* direction for the images along the testing paths. **g** Orientation measurement errors in the *y* direction for the images along the testing paths

Next, we conduct orientation measurement experiments to verify the high performance of the LCE. As shown in Fig. 5a, the LCE is fixed on a high-precision three-axis turntable. Photons from a light source (white light with a rich spectrum) pass through a collimator and form digital images at the LCE (see Supplementary Note 2 for cases of nonparallel light). The turntable can be rotated more than 120° to simulate a very precise target relative motion across a wide field (Fig. 5b). The determined orientation vector of the target is compared with the given information of the turntable for accuracy assessment (see Supplementary Note 4). Calibration is preperformed because of the complex coupling factors, such as the refraction error of the image sensor protective glass and the installation error of the subeye aperture array (see Supplementary Note 5 for details).

In the static target measurement experiment, the turntable is stabilized at certain positions. Taking one position as an example, we sample 100 images, and the result shows that the target orientation determined by the LCE is very precise, with a standard deviation of less than 0.0001° (1σ) (Fig. 5c, d). Different from the mesh positions used for calibration, 8 testing paths, as shown in Fig. 5e, are selected from the full FOV for accuracy evaluation. Along the paths, an image is collected at each step of 0.5° and then analyzed. The results show that the LCE achieves an orientation measurement accuracy of 0.0023° (3σ) in the *x* direction and 0.0028° (3σ) in the *y* direction across a wide FOV of 120° (Fig. 5f, g). This experiment proves that a static point-like target at an arbitrary position in the FOV of the LCE can be perceived with high accuracy.

**Fig. 6:**
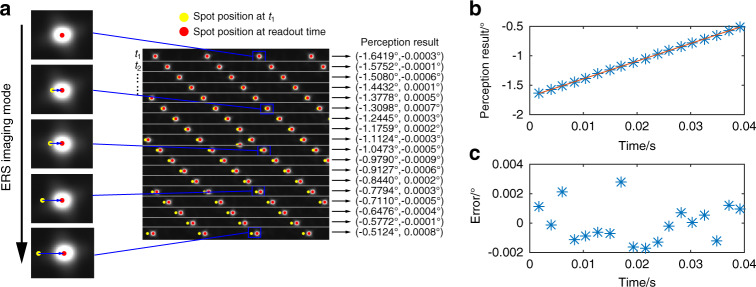
Experimental results for high-dynamic target orientation measurement. **a** Image of the target in the central sub-FOV. **b** Orientation measurement results in the *x* direction over time. **c** Orientation measurement errors in the *x* direction over time

Then, we conduct a high-dynamic target orientation measurement experiment. The turntable is rotated at a high speed of ~30°/s. The exposure time of the LCE is 20 ms, and the readout time interval between adjacent exposure rows is 20 μs. Taking the sampled image when the target is in the central sub-FOV as an example (Fig. 6a), the spots would coincide with the matched apertures (labeled by yellow circles in Fig. 6a) if the readout time is the same for the entire image. Due to the ERS technique, the readout time is different for different exposure rows, and the positions of the spots contain the hyperframe target motion information. The result shows that in this high-dynamic case, the measurement error of the LCE is 0.0045°(3σ) (Fig. 6b, c) (see Supplementary Note 6). Since the spots are distributed on 18 different exposure rows, we achieve an update rate 18 times higher than the frame rate. The frame rate of the image sensor in the experiment is 20 Hz; thus, the update rate is 360 Hz, which is more than threefold higher than that measured in the ommatidia of fast-flying insects (ref. ^39^). Our approach is not limited to this because the update rate can be significantly improved by arranging the apertures more densely and improving the frame rate of the image sensor. More results for the dynamic target measurement can be seen in Supplementary Note 7. This experiment proves that a dynamic target with an arbitrary path in the FOV of the LCE can be perceived in real time with high accuracy.

## Discussion

A lensless compound eye microsystem is developed for target orientation measurement with high performance, which consists of a planar coded subeye aperture array and a planar multiplexing image sensor. Both simulation and experimental results show that the LCE has the advantages of high resolution, a wide FOV, a hyperframe update rate, and a small and simple structure, even though in usual cases these capacities are restricted with each other. The work provides a promising approach for achieving optical measurements with comprehensive high performance and can be easily adapted to meet the requirements of various real-time motion measurement applications. A higher resolution can be achieved by choosing a longer focal length. A wider FOV can be realized by increasing the size of the subeye aperture array if the system volume limit is not strict. A higher update rate can be achieved by arranging the subeye apertures more densely, which also results in better accuracy because the number of spots increases. In addition, the LCE can simultaneously measure multiple sparse targets as long as the spots of these targets are distinguishable (see Supplementary Note 8). It can also adapt to diverse targets with different intensities due to the well-established technology of image sensors to adjust the exposure time, gain, and other parameters (see Supplementary Note 9).

**Fig. 7:**
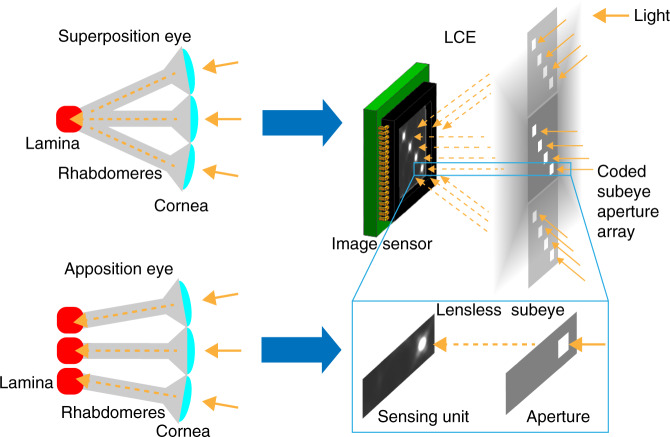
Relationship between LCE and superposition eyes, apposition eyes

The LCE can be regarded as a novel compound eye microsystem integrating the architectures of superposition eyes and apposition eyes (ref. ^8^). Different regions of the coded subeye aperture array are projected to the single multiplexing image sensor, which is consistent with superposition eyes. Target-orientation measurement is achieved by matching a single imaging spot and a single aperture, which is consistent with apposition eyes (see Fig. 7). One main limitation of the work is that we only achieve target orientation measurement without obtaining the distance information. However, the LCE is essentially a multiaperture system, which has the potential to perform distance measurement. In addition, several LCE instruments can be used together for three-dimensional target positioning in the future. The other limitation is that the easy-to-manufacturing MEMS aperture array causes poor image quality. A coded microlens array will be developed in the future to enhance the image quality, improve the system performance and expand the application scenarios.

## Materials and methods

### Spot profile simulation based on Fresnel–Kirchhoff diffraction

The spot profile formed by a subeye aperture is a diffraction image with its intensity distribution following the Fresnel–Kirchhoff diffraction formula (ref. ^40^). For near-parallel light, the intensity distribution becomes (see Supplementary Note 10 for derivation)

3 $$\begin{array}{l}\tilde E\left( P \right) = \frac{A}{{i\lambda }}\mathop {\iint}\limits_\Sigma {\exp \left[ {ik\left( {x_0\cos \alpha + y_0\cos \beta } \right)} \right]}\\\qquad\quad\;\;\, \frac{{\exp \left( {ikr} \right)}}{r}\left[ {\frac{{\cos \left( {{{{\boldsymbol{n}}}},{{{\boldsymbol{r}}}}} \right) - \cos \left( {{{{\boldsymbol{n}}}},{{{\boldsymbol{l}}}}} \right)}}{2}} \right]d\sigma\end{array}$$

where *A* is a constant related to the intensity of the light source, *λ* is the wavelength of the light wave, *k* is the wavenumber, *x*_0_ and *y*_0_ are the horizontal and vertical coordinates of the integral surface element d*σ* in the aperture region, *α* and *β* denote two direction cosines of the incident light, the meanings of vectors ***n***, ***r*** and ***l*** shown in Fig. 2b, and *r* is the norm of ***r***. According to this equation, the profiles formed by subeye apertures from different regions can be simulated and analyzed. Here, we use the half width of the region that covers 80% of the energy of the intensity distribution to evaluate the energy concentration of the spot. The wavelength is set as 531 nm (see Supplementary Note 3 for analysis of wavelength), and the *l*_*p*_ for the profiles in Fig. 2c is 0.055 mm, 0.065 mm, 0.075 mm, 0.095 mm, 0.115 mm, and 0.135 mm and in Fig. 2d is 0.176 mm, 0.186 mm, 0.196 mm, 0.216 mm, 0.236 mm, and 0.256 mm. *l*_*v*_ for these profiles is 0.075 mm.

### Manufacturing of the subeye aperture array

The coded subeye aperture array has a simple manufacturing process, and the MEMS processing technology typical for mask plate manufacturing is adopted as shown in Fig. 3b. The substrate of the MEMS aperture array is quartz glass, and a layer of chromium with a thickness of 100 nm is plated on the surface of the glass. To protect the coating, chromium oxide with a thickness of 10 nm is plated on the chromium layer. Through the MEMS mask fabrication process, the photoresist is coated on the chromium-plated quartz substrate and exposed by the laser or electron beam according to the design of the coded subeye aperture array. After the exposed photoresist is removed, the chromium layer and chromium oxide layer are exposed and removed by etching. At this point, the etched part allows light to pass through, while the other part rejects light. After resist stripping, the required coded subeye aperture array can be obtained.

### LCE performance analysis

The sub-FOV in the *x* direction of a subeye aperture can be calculated by

4 $$\begin{array}{l}\left[ FOV_{x1},FOV_{x2} \right] = \left[ {\arctan \left( {\frac{{2x_a - l_{sensor}}}{{2h}}} \right)}\right.,\\\left.\qquad\qquad\qquad\qquad\;\arctan \left( {\frac{{2x_a + l_{sensor}}}{{2h}}} \right) \right]\end{array}$$

where *l*_*sensor*_ is the length of the image sensor. Here, *l*_*sensor*_ = ~4.9 mm, *h* = 7 mm, and *x*_*a*_ can be taken from the interval [−15 mm, 15 mm]. The calculation of the sub-FOV in the *y* direction is similar. In cases of dense aperture distribution, there are large overlapping fields between adjacent apertures. Thus, multiple spots can be formed when one target enters the FOV of the LCE, providing the possibility for coding.

The angular resolution (*Δ*α) of the LCE for orientation measurement meets

5 $$h \times \left[ {\tan \left( {\alpha + \Delta \alpha } \right) - \tan \left( \alpha \right)} \right] = \Delta l$$

Thus,

6 $$\Delta \alpha = \arctan \left[ {\frac{{\Delta l}}{{\left( {1 + \tan ^2\alpha } \right) \times h + \tan \alpha \times \Delta l}}} \right]$$

where *α* is the incident angle of the light and Δ*l* is the minimum displacement of the spot that can be resolved by the image sensor. Δ*l* can be determined by estimating the limit of subpixel centering precision based on the spot profile simulation (ref. ^38^). Combined with the long focal length (*h*) of the microsystem, LCE enables high orientation measurement resolution.

The maximum update rate *U*_*max*_ of our approach can be estimated by (ref. ^41^)

7 $$U_{\max } = \left[ {\frac{{l_{sensor}}}{{l_{a\min }}}} \right] \cdot n_{fps}$$

where *n*_*fps*_ is the frame rate of the image detector and *l*_*amin*_ is the minimum length of the spot that can be formed. Here, we select 37% of the maximum intensity as the extracting threshold and assume that the length of the extracted spot region is *l*_*a*_. The *l*_*amin*_ of the spot for the vertically incident light (Fig. 4e) is 0.050 mm, and that for the 60° incident light (Fig. 4f) is 0.164 mm. Thus, the maximum update rate can be ~30–100 times higher than the frame rate.

## Supplementary information

Supplementary Information

## Data Availability

The datasets generated during the current study are available from the corresponding author upon reasonable request.
