# Supplementary material for: A wide-field and high-resolution lensless compound eye microsystem for real-time target motion perception
Source: Microsyst Nanoeng. 2022 Jul 22;8:83. doi: 10.1038/s41378-022-00388-w (PMC9304386; doi:10.1038/s41378-022-00388-w)
Supplement: Supplementary file 1 — Supplementary Information [file 41378_2022_388_MOESM1_ESM.docx]

Supplementary Information

**A wide-field and high-resolution lensless compound eye system for real-time target motion perception**

Li Zhang^1,2,3,4^, Haiyang Zhan^1,2,3,4^, Xinyuan Liu^1,2,3^, Fei Xing^1,2,3,*^, Zheng You^1,2,3,*^

^1^ Department of Precision Instrument, Tsinghua University, Beijing 100084, China.

^2^ State Key Laboratory of Precision Measurement Technology and Instrument, Tsinghua University, Beijing 100084, China

^3^ Beijing Advanced Innovation Center for Integrated Circuits Beijing, 100084, China.

^4^ Li Zhang and Haiyang Zhan contributed equally to this work.

* Contacting e-mail: xingfei@mail.tsinghua.edu.cn, yz-dpi@mail.tsinghua.edu.cn

**Supplementary Table 1.** Characteristics of recently reported compound eye measurement systems

| **Ref.** | **Year** | **Name** | **FOV** | **Angular resolution** | **Update rate** | **Volume** |
| --- | --- | --- | --- | --- | --- | --- |
| [1] | 2003 | TOMBO | 157.4°×157.4° | 0.44° | \ | 5 mm× 5mm×50 μm (optical system only) |
| [2] | 2012 | \ | 360°×  90° | 0.35° | 25fps | SΦ129mm/30mm  (hemisphere) (optical system only) |
| [3] | 2013 | \ | 160° | 1.1° | \ | 14.72 mm×14.72 mm×R6.96 mm (optical system only) |
| [4] | 2013 | CurvACE | 180°×  60° | 4.2° | 300Hz | 13 mm×12 mm×10 mm |
| [5] | 2017 | SCECam | 122.4°×360° | 0.15° | 35fps | 40 mm×40 mm×80 mm |
| [6] | 2017 | \ | 40° | 0.08° | 75fps~1000fps | 60 mm×60 mm×50 mm |
| [7] | 2018 | \ | 68° | ～0.2° | \ | Φ3.4 mm×1.4 mm (optical system only) |
| [8] | 2020 | \ | 73° | ～1.5° | 30fps | 5.1 mm×5 mm×0.74 mm (optical system only) |
| [9] | 2020 | \ | 150° | ～1.5° | \ | \ |
| [10] | 2020 | \ | 120°×  120° | θ:0.5951° φ:0.6748° | \ | 100 mm×100 mm×100 mm |

Note:

There is no clear definition and direct calculation of the angular resolution in many references. It is calculated as follows: (1) if the image resolution is provided, we find the equivalent focal length, and calculate the equivalent angular resolution according to the two parameters; (2) If the field of view and the image resolution are provided, the field of view angle corresponding to a single signal sensing unit is used as the angular resolution.

**References**

1. [Jun T, Rui S, Yoshiro K, et al. Color imaging with an integrated compound imaging system. Opt. Express, 2003, 11(18): 2109-2117](https://doi.org/10.1364/OE.11.002109)
2. [Hossein A; Vladan P; Tugce T, et al. A spherical multi-camera system with real-time omnidirectional video acquisition capability. IEEE trans. consum. electr., 2012, 58(4): 1110 - 1118](https://doi.org/10.1109/TCE.2012.6414975)
3. [Young MS, Xie Y, Viktor M, et al. Digital cameras with designs inspired by the arthropod eye. Nature., 2013, 497: 95-99](https://doi.org/10.1038/s41377-020-0261-8)
4. [Dario F, Ramon PC, Stéphane V, et al. Miniature curved artificial compound eyes. PNAS, 2013, 110(23): 9267-9272](https://doi.org/10.1073/pnas.1219068110)
5. [Shi CY, Wang YY, Liu CY, et al. SCECam: a spherical compound eye camera for fast location and recognition of objects at a large field of view. Opt. Express, 2017, 25(26): 32333-32345](https://doi.org/10.1364/OE.25.032333)
6. [Pang, K., Fang, F. Z., Song, L., Zhang, Y. & Zhang, H. Y. Bionic compound eye for 3D motion detection using an optical freeform surface. J. Opt. Soc. Am. B-Opt. Phys. 34, B28-B35,2017](https://doi.org/10.1364/josab.34.000b28)
7. [Keum D, Jang KW, Jeon DS. Xenos peckii vision inspires an ultrathin digital camera. Light Sci. Appl., 2018, 7(1): 80](https://doi.org/10.1038/s41377-018-0081-2)
8. [Kim K, Jang KW, Ryu JK. Biologically inspired ultrathin arrayed camera for high-contrast and high-resolution imaging. Light Sci. Appl., 2020, 9(1): 28](https://doi.org/10.1038/s41377-020-0261-8)
9. [Kogos LC, Li YZ, Liu JN, et al. Plasmonic ommatidia for lensless compound-eye vision. Nat. Commun., 2020, 11(1): 1637](https://doi.org/10.1038/s41467-020-15460-0)
10. [Ma MC, Li H, Gao XC, et al. Target orientation detection based on a neural network with a bionic bee-like compound eye. Opt. Express, 2020, 28(8): 10794-10805](https://doi.org/10.1364/OE.388125)

**Supplementary Table 2.** Parameters of the lensless compound eye (LCE) microsystem

| **Name** | **Value** |
| --- | --- |
| FOV | cone FOV of 120° |
| Pixel number of the image sensor (used) | 2048×2048 |
| Pixel size of the image sensor | 2.4 μm |
| Focal length of the system | 7 mm |
| Size of the coded subeye aperture array | Φ30 mm |
| Size of a sub-region | 4.915 mm |
| Number of the sub-regions | 37 |
| Number of the apertures | 1026 |
| Volume of the entire instrument | 32 mm×36 mm×28.3 mm |
| Weight | 44.4 g |
| Power consumption | ~1.1 W |

**Supplementary Note 1.** Perception algorithm

The basic principle for target motion perception is described in the **Principle of high-performance LCE** in the manuscript. The process of the perception algorithm is shown in **Fig. S1**. We first set up a template for the designed coded subeye aperture array, then carry out correlative operation on the collected images. According to the location of the maximum associated peak, the corresponding relationship between the subeye apertures and the spot centroid positions in digital images can be determined, and then the accurate orientation vector of the target can be calculated according to **Eq. (1)** and **Eq. (2)**. After necessary correction (form calibration), we output the perception results.

**Fig. S1** Process of the perception algorithm.

The template for the designed coded subeye aperture array is shown in **Fig. S2 a**. The acquired image of a target at a certain location is shown in **Fig. S2 b**. We correlate the two graphs. Due to different coding rules at different positions, the acquired image only fully matches a certain region of the LCE template, where corresponds to the maximum correlation peak, as shown in **Fig. S2 c**.


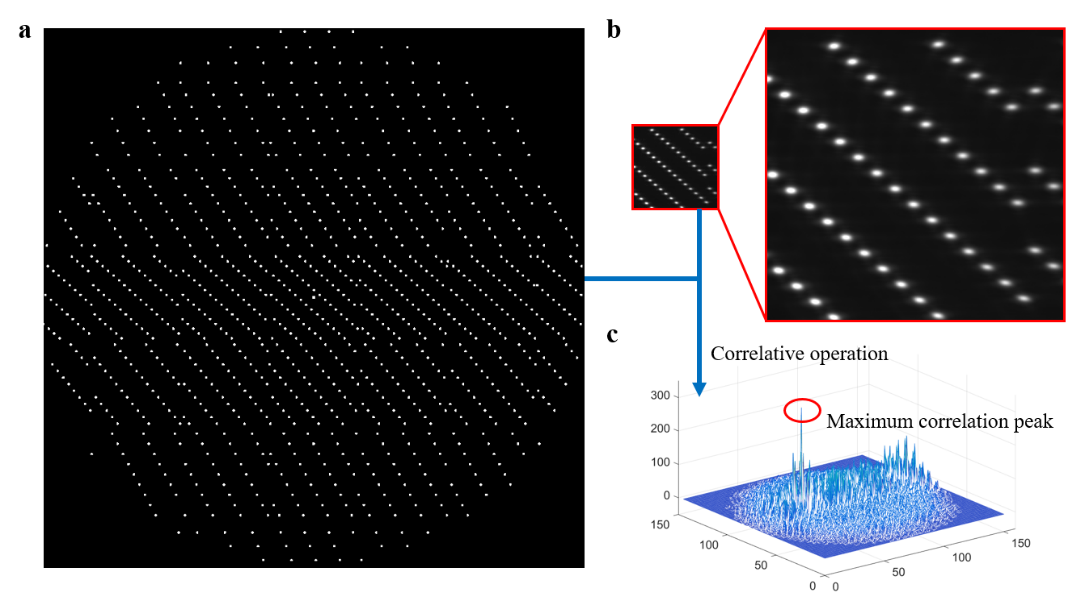


**Fig. S2** **a** Template of the coded subeye aperture array. **b** Acquired image; **c** Result of the correlation operation.

According to the position of the maximum correlation peak, the area of the coded subeye aperture array matching the acquired image can be determined, as shown in **Fig. S3 a**. According to the principle of shortest Euclidean distance, the one-to-one correspondence between the light spot positions in the acquired image and the centers of the coded subeyes is obtained, as shown in **Fig. S3 b**. Finally, according to the centroid positions of the light spots in the acquired image and the center positions of the matched coded subeyes of LCE, **Eq. (1)** and **Eq. (2)** can be used to calculate the orientation perception result.


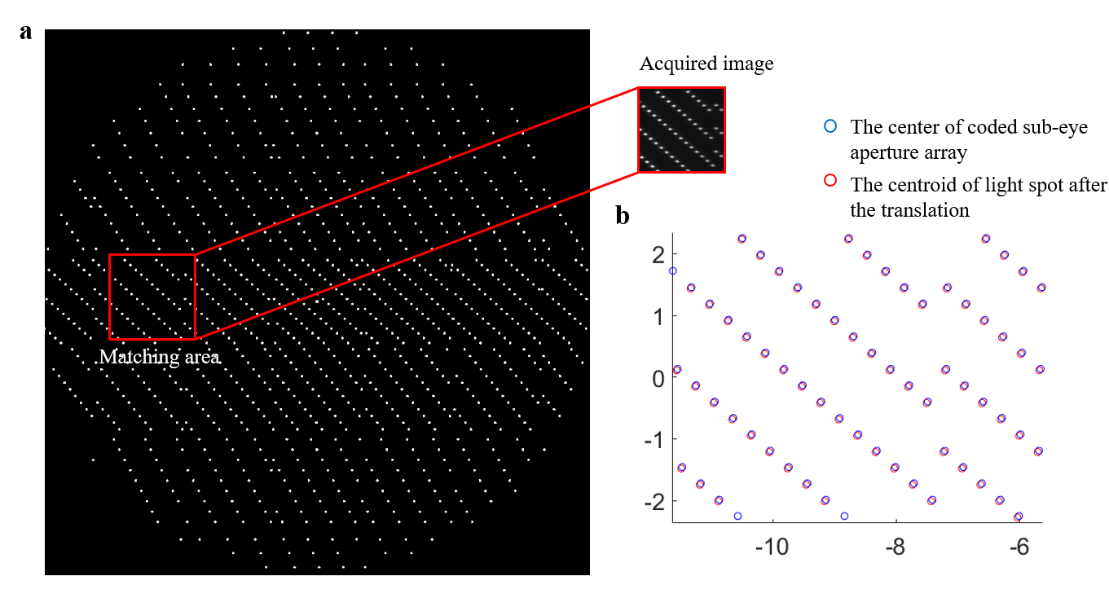


**Fig. S3** **a** Matching result of the acquired image with the coded subeye aperture array. **b** Comparison between the spot positions in the image and the center positions of the matched coded subeyes.

**Supplementary Note 2.** Measurement model of the LCE for non-parallel light

The perception process in the paper is for parallel light, but the result is slightly different when the incident beam is non-parallel. Here, we analyze the difference between the two cases in a simplified way in one dimension, as the conclusion is the same in two dimensions.

When perceived target is far away from the system, the light emitted by the target reaches the LCE and can be considered as a parallel beam, such as the sun. At this time, after the matching of the spots and the subeye apertures is completed, the calculating orientation vector can be determined from each pair of spot and aperture. We then take the average of the orientation vectors for higher accuracy. The process is shown in **Fig. S4 a**.


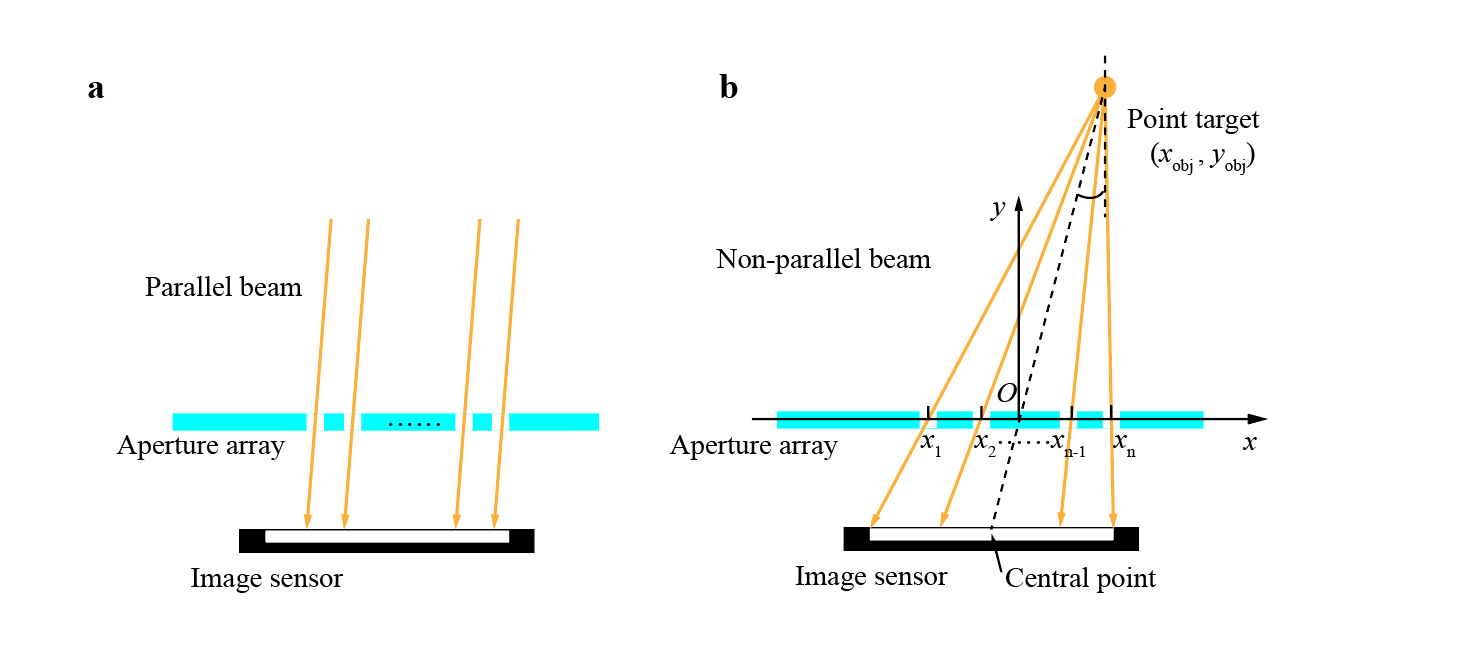


**Fig. S4** **a** Measurement for parallel light. **b** Measurement for non-parallel light.

When a perceived target (simplified as a point target) is close to the system, there are different incident angles for different subeye apertures, and the difference between the incident angles is even larger than the measurement error of the system, as shown in **Fig. S4 b**. In this case, the line between the center of the image sensor and the point target is taken as the true orientation vector. We assume the intersection between the vector and the aperture array as the origin, establish the coordinate system as shown in **Fig. S4 b**, set the coordinate of the point target as (*x_obj_*, *y_obj_)*, and then the real perception result should be

$A_{ture}=\arctan\left(\frac{x_{obj}}{y_{obj}}\right)$ . (S1)

It can be found that the incident light vectors passing through different subeyes are distributed on both sides of the real result. In the perception algorithm, the final result is the average value of the incident light vectors corresponding to all the spots in the image. Therefore, the calculation result of the algorithm is

$A_{measure}=\frac{\sum_{i=1}^{n} \arctan\left(\frac{x_{obj}-x_{i}}{y_{obj}}\right)}{n}$ . (S2)

The error is

$Error=A_{measure}-A_{ture}=\frac{\sum_{i=1}^{n} \arctan\left(\frac{x_{obj}-x_{i}}{y_{obj}}\right)}{n}-\arctan\left(\frac{x_{obj}}{y_{obj}}\right)$ . (S3)

The coded subeye aperture array has been designed in the **LCE design and optimization** of the manuscript. When the position of the point target is known, we can deduce the incident angle of the light (*A_ture_*) and the coordinates of subeyes that can image on the image sensor. By putting this data into the calculation formula of *Error*, the influence of the point target’s spatial position on the measurement result can be obtained. As can be seen from **Fig. S5 a**, in the *x* direction, when the vertical distance between the point target and LCE is farther than 4m, the measurement error caused by the non-parallel light is less than the measurement accuracy in the *x* direction. As can be seen from **Fig. S5 b**, in the *y* direction, when the vertical distance between the point target and the LCE is farther than 8m, the measurement error caused by the non-parallel light is less than the measurement accuracy in the *y* direction.

LCE aims at the precise measurement for parallel light or nearly parallel light. When the point target is more than 10m away from LCE in vertical distance, the non-parallel light generated by the point target has little influence on the measurement accuracy of the system. This distance is easy to reach in collision-free navigation and formation of UAV, vision-controlled directional navigation and other applications.


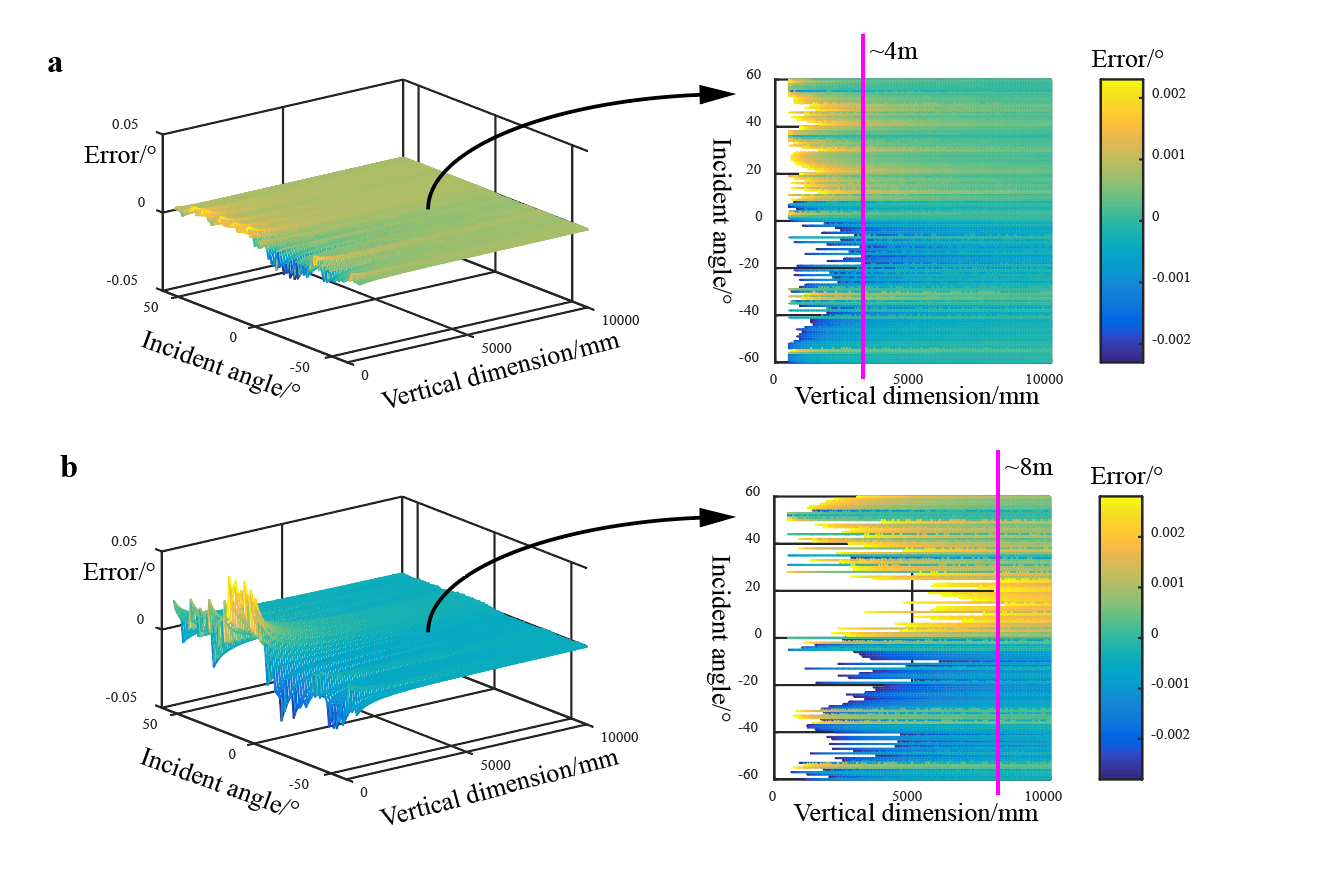


**Fig. S5** Influence of non-parallel light on measurement accuracy. **a** The left side represents the influence of the point target’s spatial position on the measurement accuracy in the *x_s_* direction, and the right side selects data within the *x_s_* measurement accuracy (0.0023 (3σ)) for observation. **b** The left side represents the influence of the point target’s spatial position on the measurement accuracy in the *y_s_* direction, and the right side selects the data within the *y_s_* measurement accuracy (0.0028 (3σ)) for observation.

**Supplementary Note 3.** Analysis of the detection wavelength of LCE

The judgment basis of aperture optimization is given in the section **Spot profile simulation based on the Fresnel-Kirchhoff diffraction**. According to **Eq. (3)**, the simulation of the diffraction can be analyzed under light with different angles, different wavelengths, different light intensities and different aperture sizes.

The optimal aperture size varies with the incident angle and the wavelength of the optical target. The optimal aperture sizes under different wavelengths for the vertically incident light and the 60° incident light are shown in **Fig. S6**.


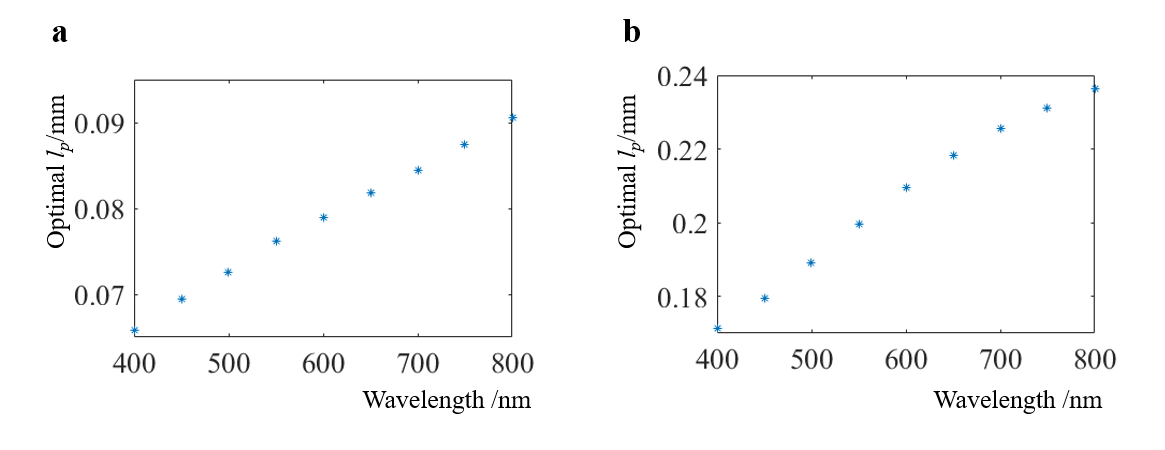


**Fig. S6 a** Optimal aperture size at different wavelengths for the vertically incident light. **b** Optimal aperture size at different wavelengths for the 60° incident light.

For the perceived target containing a light spectrum, its image acquired by LCE is the superposition of the diffraction spots formed by the light with different wavelengths passing through the coded subeye aperture array. The size of the coded subeye aperture can only be optimized for the light with a particular wavelength. The peak sensitivity of human eyes is in the blue-green part of the spectrum (507nm) in scotopia vision, and in the yellow-green part of the spectrum (555nm) in photopic vision [1]. Biological eyes have similar sensitivity to light, and the spectral characteristics of compound eyes of different insects are slightly different. Therefore, the average value (531nm) of spectral sensitivity peak of human eyes under scotopia and photopic vision is selected as the wavelength for optimization. The optimal wavelength corresponding to different incident angles is shown in **Supplementary Table 3**.

When the aperture size is determined, the diffraction effects for the light with different wavelengths are slightly different, as shown in the **Fig. S7**. In the case of incident light with white light, the diffraction pattern can be approximated as the superposition of several patterns in **Fig. S7**. Through experiments (see **Orientation Measurement for Static and Dynamic Targets**), it can also be seen that LCE is robust to the wavelength of incident light.

**Supplementary Table 3.** Results for aperture size optimization

| **Incident angle /°** | **Optimal *l_p_* /mm** |
| --- | --- |
| 0 | 0.0750 |
| 5 | 0.0755 |
| 10 | 0.0765 |
| 15 | 0.0785 |
| 20 | 0.0810 |
| 25 | 0.0855 |
| 30 | 0.0905 |
| 35 | 0.0980 |
| 40 | 0.1080 |
| 45 | 0.1200 |
| 47 | 0.1265 |
| 50 | 0.1365 |
| 52 | 0.1450 |
| 55 | 0.1615 |
| 57 | 0.1730 |
| 60 | 0.1960 |


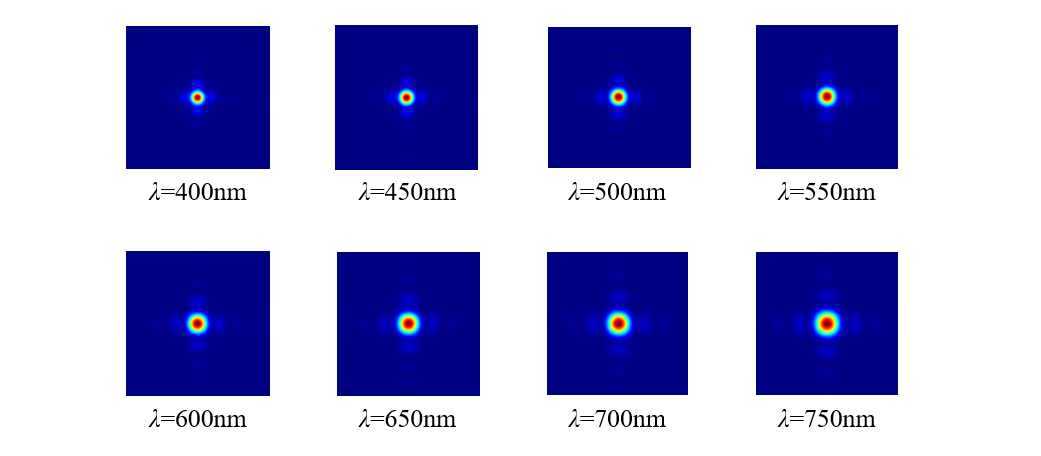


**Fig. S7** The diffraction effect of light waves with different wavelengths when *l_p_*=0.075mm at vertical incidence.

For the perceived target whose spectrum are known, the aperture distribution can be optimized according to its spectrum. This paper only introduces an optimization method and process.

Some simulated diffraction spot profiles and intensity distribution curves are shown in **Fig. S8** and **Fig. S9**.


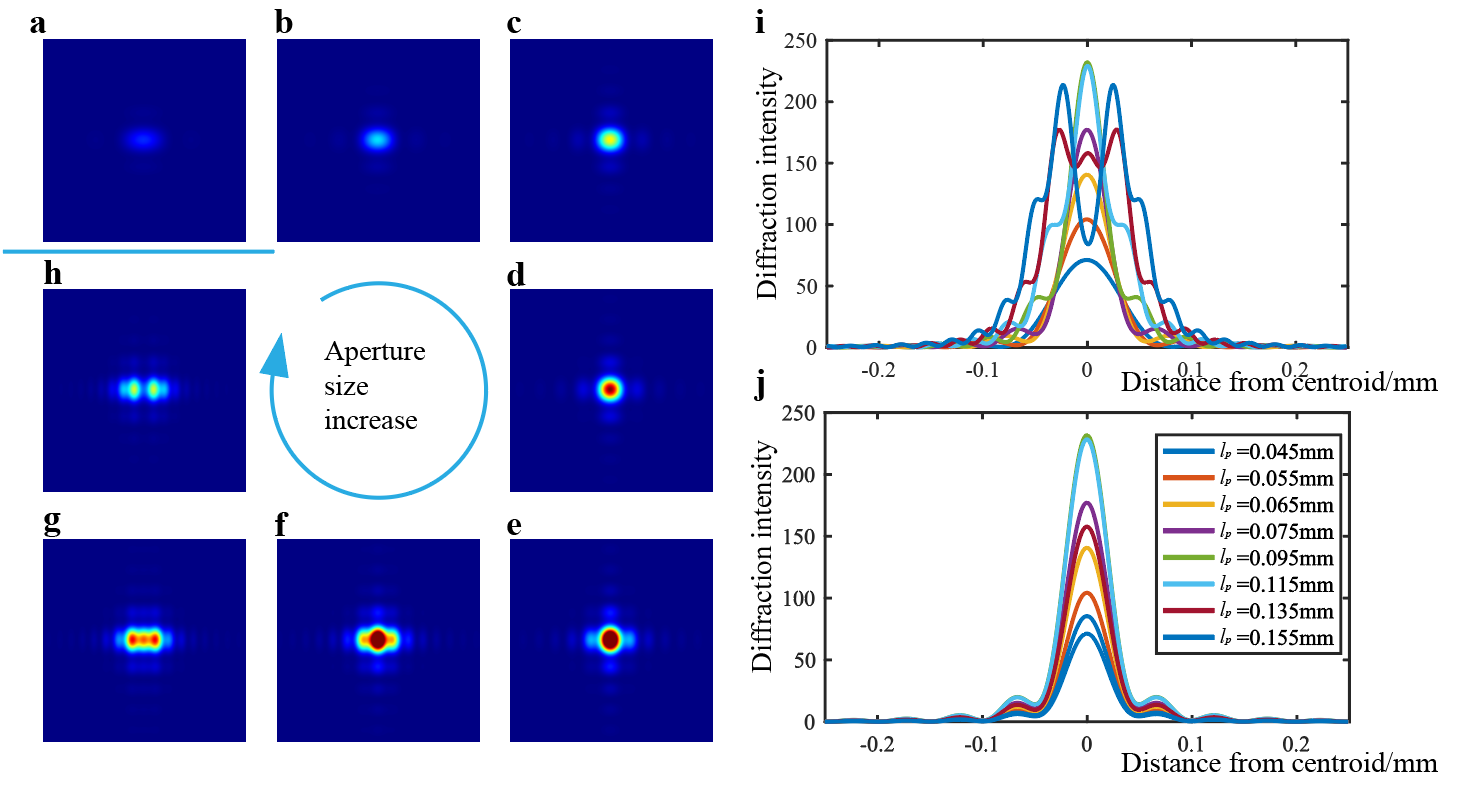


**Fig. S8** Simulated diffraction spot profiles and intensity distribution curves formed by changing *l_p_* of the aperture when the light is vertically incident, with the focal length of 7mm and the wavelength of 531nm. **a** ~ **h** Spot profiles with *l_p_* =0.045mm, *l_p_* =0.055mm, *l_p_* =0.065mm, *l_p_* =0.075mm, *l_p_* =0.095mm, *l_p_* =0.115mm, *l_p_* =0.135mm, *l_p_* =0.155mm, respectively. *l_v_* is fixed as 0.075 mm. **i** Intensity distribution of the spots in the *l_p_* direction corresponding to different *l_p_* of the aperture. **j** Intensity distribution of the spots in the *l_v_* direction corresponding to different *l_p_* of the aperture. Note that the vertical coordinates in **i** and **j** only represent relative intensity.


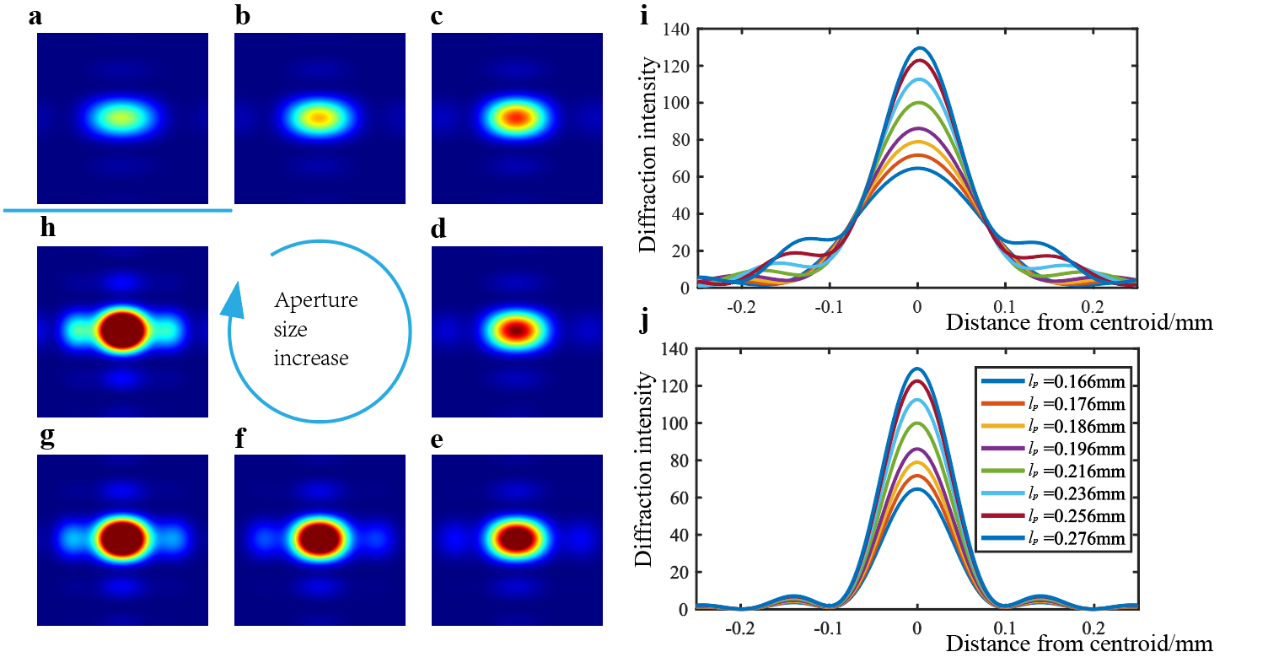


**Fig. S9** Simulated diffraction spot profiles and intensity distribution formed by changing *l_p_* of the aperture when the light is obliquely incident (60°), with the focal length of 7mm and the wavelength of 531nm. **a** ~ **h** Spot profiles with *l_p_* =0.166mm, *l_p_* =0.176mm, *l_p_* =0.186mm, *l_p_* =0.196mm, *l_p_* =0.216mm, *l_p_* =0.236mm, *l_p_* =0.256mm, *l_p_* =0.276mm, respectively. *l_v_* is fixed as 0.075 mm; **i** Intensity distribution of the spots in the *l_p_* direction corresponding to different *l_p_* of the aperture; **j** Intensity distribution of the spots in the *l_v_* direction corresponding to different *l_p_* of the aperture. Note that the vertical coordinates in **i** and **j** only represent relative intensity.

[1] *Applied Optics (4th Edition)*. (Publishing House of Electronics Industry, 2015).

**Supplementary Note 4.** Euler angle transformation in the experiment

When the turntable is at a certain angle, the coordinate system of the turntable does not coincide with the coordinate system of LCE. The angles of the outer axis and the central axis of the turntable are different from the angles calculated by LCE in the *x* direction and in the *y* direction, and there is a transformation relationship.

Suppose a reference coordinate system is *o*_0_*x*_0_*y*_0_*z*_0_, and *o*_1_*x*_1_*y*_1_*z*_1_ is a new coordinate system transformed from *o*_0_*x*_0_*y*_0_*z*_0_ by the Euler rotating mode of *y-x* with the angle (*θ*_2_, *θ*_1_). The transformation matrix between the reference coordinate system and the target coordinate system is

$T= \left[\begin{matrix} C_{1} & S_{1}S_{2} & S_{1}C_{2} \\ 0 & C_{2} & -S_{2} \\ -S_{1} & C_{1}S_{2} & C_{1}C_{2} \end{matrix}\right]$ , (S4)

where *C*_1_=cos(*θ*_1_), *S*_1_=sin(*θ*_1_), *C*_2_=cos(*θ*_2_), *S*_2_=sin(*θ*_2_).

The LCE coordinate system is set as *o_s_x_s_y_s_z_s_*. The origin of the coordinate system is located at the center of the image sensor. The *x_s_* axis and the *y_s_* axis are along the directions of the row and column of the image sensor respectively, and the *z_s_* axis is along the normal direction of the image sensor. In the verification experiment of LCE, the coordinate system of the turntable is set as *o_t_x_t_y_t_z_t_* with the installation plane center as the origin, and the three rotating axes are set as the *x_t_*, *y_t_*, *z_t_*, respectively. We install LCE onto the turntable and make the two coordinate system coincide with each other, and make the light from the source enter LCE along the *z_s_* axis. At this time, the unit vector of the light in *o_s_x_s_y_s_z_s_* can be expressed as $\vec{A}$= (0, 0, -1). After rotating the turntable according to the *y-x* Euler angle (*θ*_2_, *θ*_1_), the unit vector in the new *o_s_x_s_y_s_z_s_* becomes

$\overrightarrow{A'}=\overrightarrow{A}T=\left[\begin{matrix} \sin\left(\theta_{1}\right) & -\cos\left(\theta_{1}\right)\sin\left(\theta_{2}\right) & -\cos\left(\theta_{1}\right)\cos\left(\theta_{2}\right) \end{matrix}\right]$ . (S5)

According to the vector $\vec{\text{A'}}$, the theoretical incident angle of the two axes can be deduced as follows:

$A_{x}=-\arctan\frac{\tan\left(\theta_{1}\right)}{\cos\left(\theta_{2}\right)}$ (S6)

$A_{y}=\theta_{2}$ (S7)

**Supplementary Note 5.** Calibration process and principle

The error of the instrument is mainly divided into the systematic error and the random error. The systematic error of the instrument is caused by the design defects and deficiencies of its working principle, system structure and other aspects. Generally, the systematic error has certainty and regularity, which can be corrected or compensated by instrument calibration. The random error is uncertain, which is generally represented by the standard deviation form statistical analysis. The systematic error of this instrument mainly comes from the following aspects:

- Focal length error. Due to installation, the distance between the coded subeye aperture array and the image sensor of the instrument is slightly different from the set value, so *h* in the **Eq. (1)** and **Eq. (2)** is inaccurate. There are systematic errors in the calculation results.
- Error of the system reference zero. In the designed model, the origin of the coded subeye aperture array and the origin of the image sensor should be on the same straight line perpendicular to the image sensor plane. But due to installation reasons, the two may not be aligned.
- Refraction error of the protective glass on the image sensor. The protective glass on the coded subeye aperture array has no effect on the image position of the target, because the apertures are below the protective glass. However, the protective glass on the surface of the image sensor affects the propagating direction of light, and then affects the imaging position of the target.

The three types of errors are shown in **Fig. S10**.


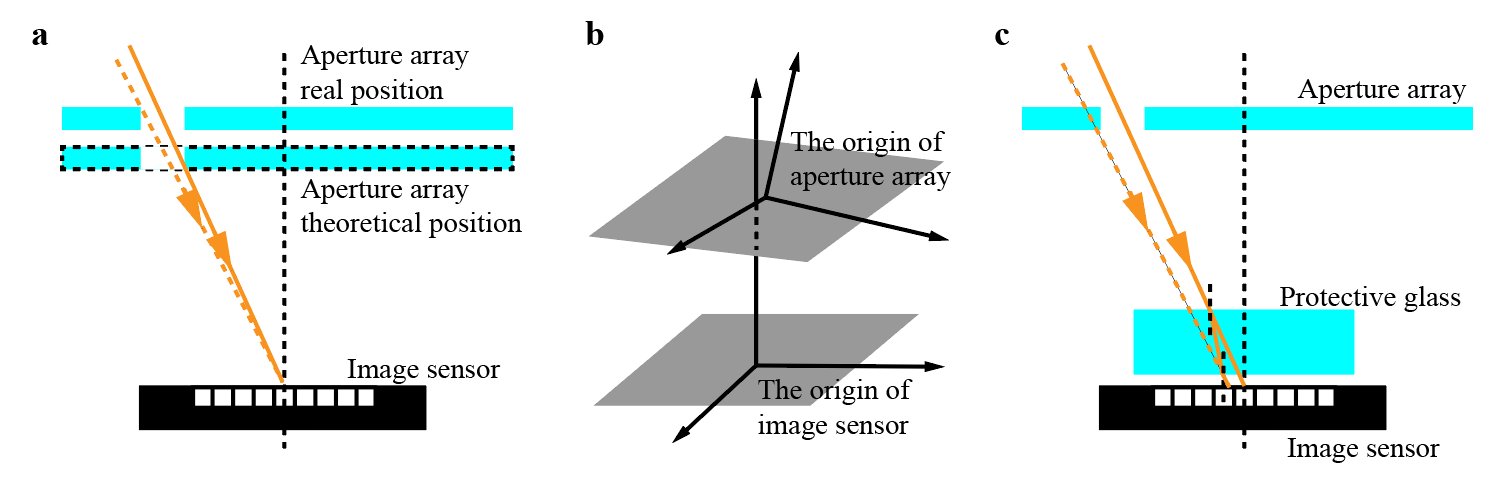


**Fig. S10 a** Focal length error. **b** Error of the system reference zero. **c** Refraction error of the protective glass on the image sensor.

These errors are coupled together and difficult to eliminate separately. In order to reduce the systematic error caused by the above factors and other factors, the system adopts the method of global calibration. First, the reference zero of the instrument needs to be determined. The instrument is fixed on a high-precision turntable. The parallel light emitted from the collimator hits the LCE and forms a series of spots on the image sensor. The determination process of the reference zero is as follows: when the turntable rotates around the internal axis, the centroid position of the central spot does not change. At reference zero, the incident ray is perpendicular to the image sensor plane. The turntable then rotates at 3° intervals within a 120° conical range. LCE images are taken at each location and the orientation vectors are calculated. By comparing the measured value with the true value from the high-precision three-axis turntable (with the precision of 0.0001°), the system generates the calibration matrix of the full FOV, as shown in **Fig. S11**. The measurement results of the system are modified according to the interpolation of the calibration matrix at the corresponding positions, and then the high accuracy measurement results are obtained.


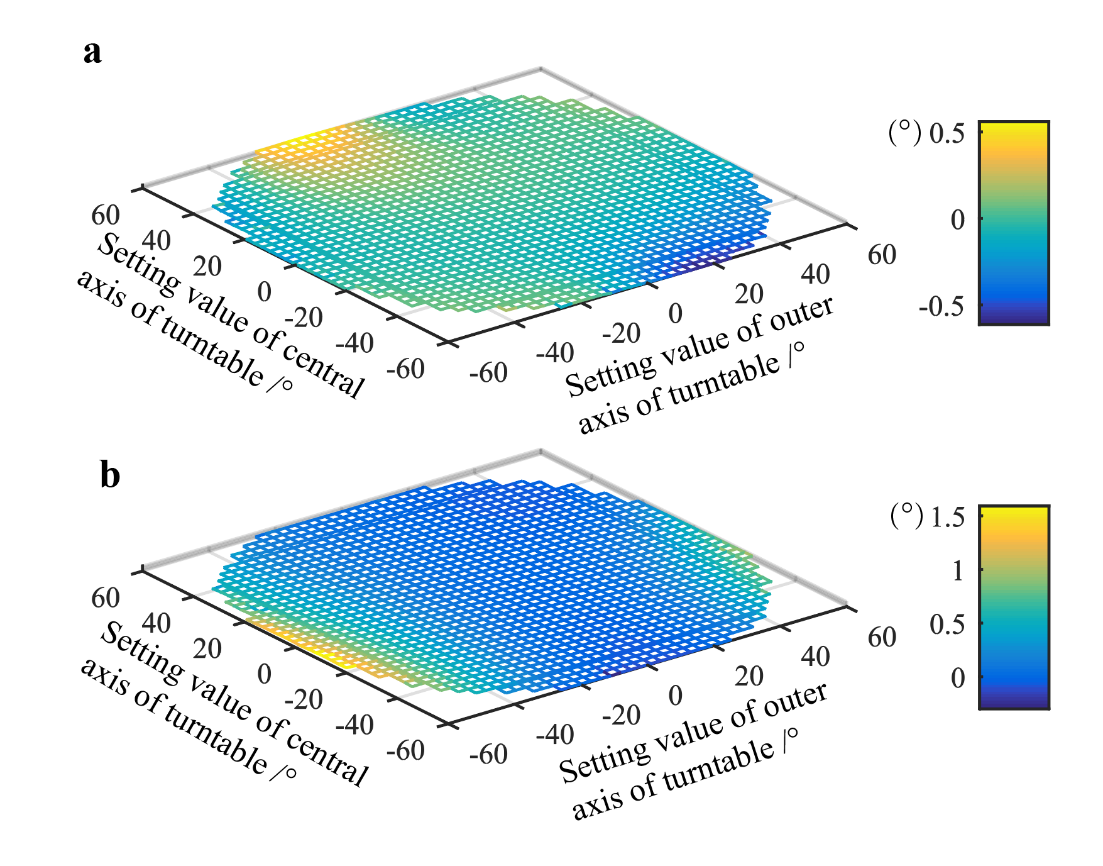


**Fig. S11** **a** Calibration matrix in the *x* direction. **b** Calibration matrix in the *y* direction.

**Supplementary Note 6.** Description of the performance related to accuracy

| **Value** | **Index name** | **Details** | **Notes** |
| --- | --- | --- | --- |
| 3.1’’ | Resolution | According to the optical measurement precision limit theory, the highest positioning precision that can be achieved by a single frame and a single spot is considered to be the resolution of the instrument. |  |
| 0.0001° | Precision | The stability of instrument measurement at the same position, reflecting the random error of instrument measurement results. |  |
| x: 0.0023°(3σ)  y: 0.0028°(3σ) | Accuracy for static target orientation measurement | Measuring accuracy from multiple spots in a single frame. | As the random error is about 0.0001°, it can be seen that the measurement error of the instrument mainly comes from systematic error, and the measurement accuracy can be improved through more intensive calibration. |
| 0.0045°(3σ) | Accuracy for dynamic target orientation measurement | Measuring accuracy from several spots on certain exposure rows in a single frame. | Due to the high speed of the turntable, it is difficult to obtain the true value. Here we set the linear fitting of the results as the true value reference. |

**Supplementary Note 7.** More results for high-dynamic target orientation measurement

Four more results for high-dynamic target orientation measurement with different rotating speeds and different target orientation vectors are shown in **Fig. S12** - **Fig. S15**.


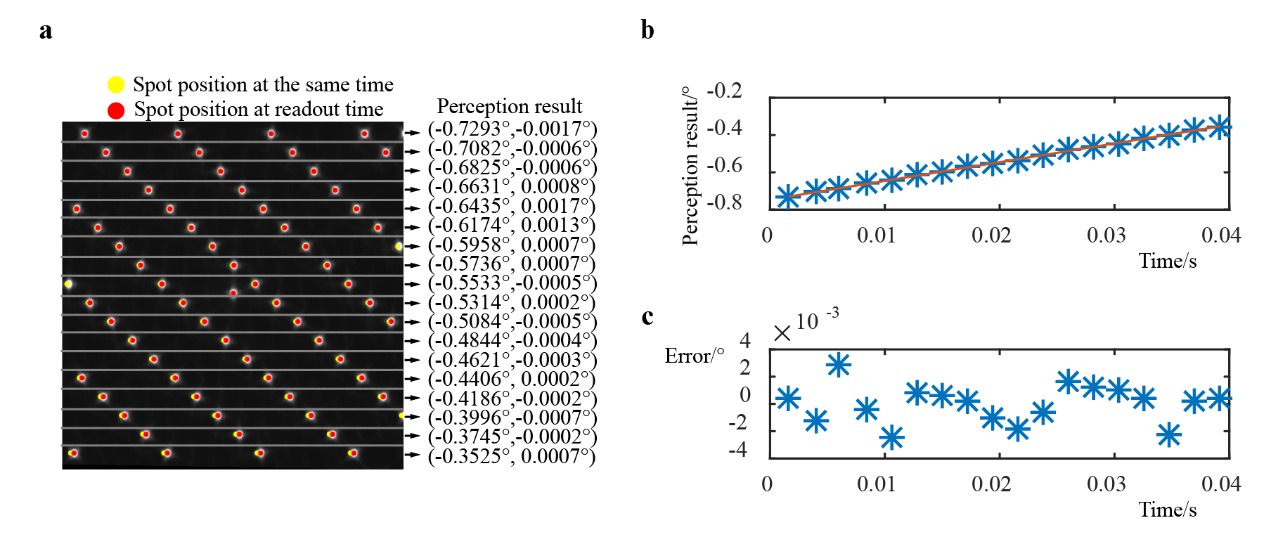


**Fig. S12** **Experiment results for high-dynamic target orientation measurement with the turntable’s rotating speed of ~10°/s.** **a** Image of the target in a central sub-FOV. **b** Orientation measurement results in the *x* direction over time. **c** Orientation measurement errors in the *x* direction over time.


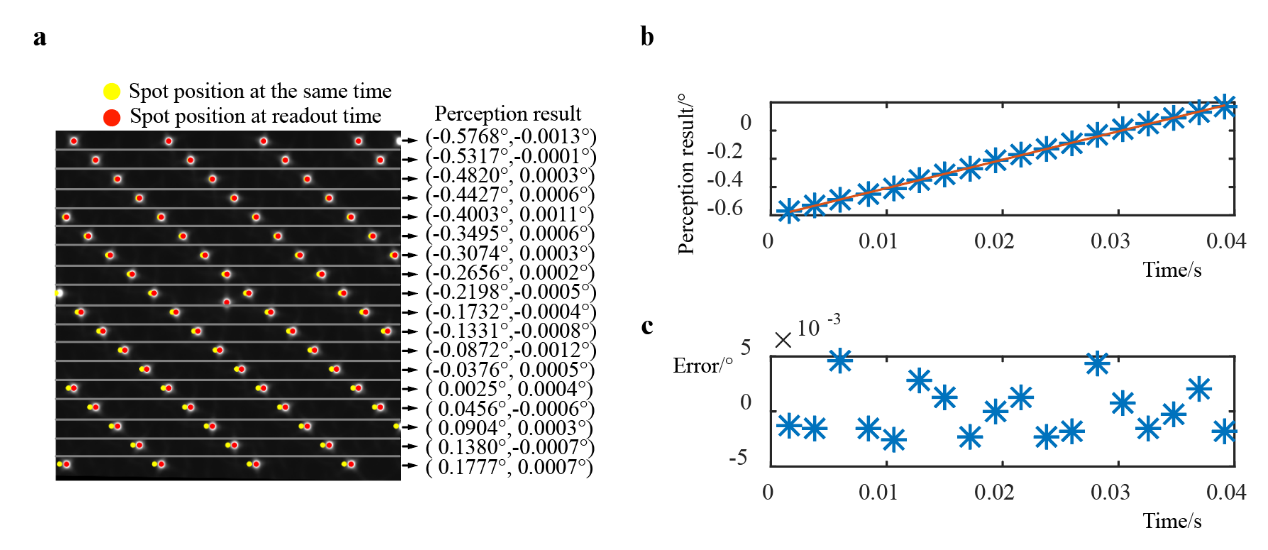


**Fig. S13** **Experiment results for high-dynamic target orientation measurement with the turntable’s rotating speed of ~20°/s.** **a** Image of the target in a central sub-FOV. **b** Orientation measurement results in the *x* direction over time. **c** Orientation measurement errors in the *x* direction over time.


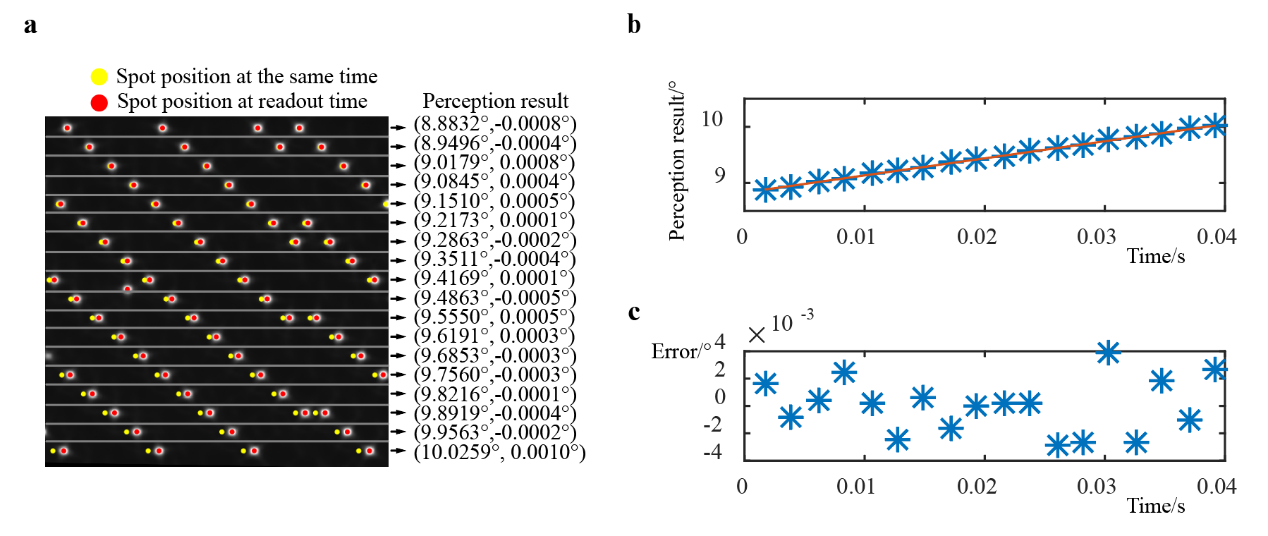


**Fig. S14** **Experiment results for high-dynamic target orientation measurement with the turntable’s rotating speed of ~30°/s.** **a** Image of the target in an arbitrary sub-FOV. **b** Orientation measurement results in the *x* direction over time. **c** Orientation measurement errors in the *x* direction over time.


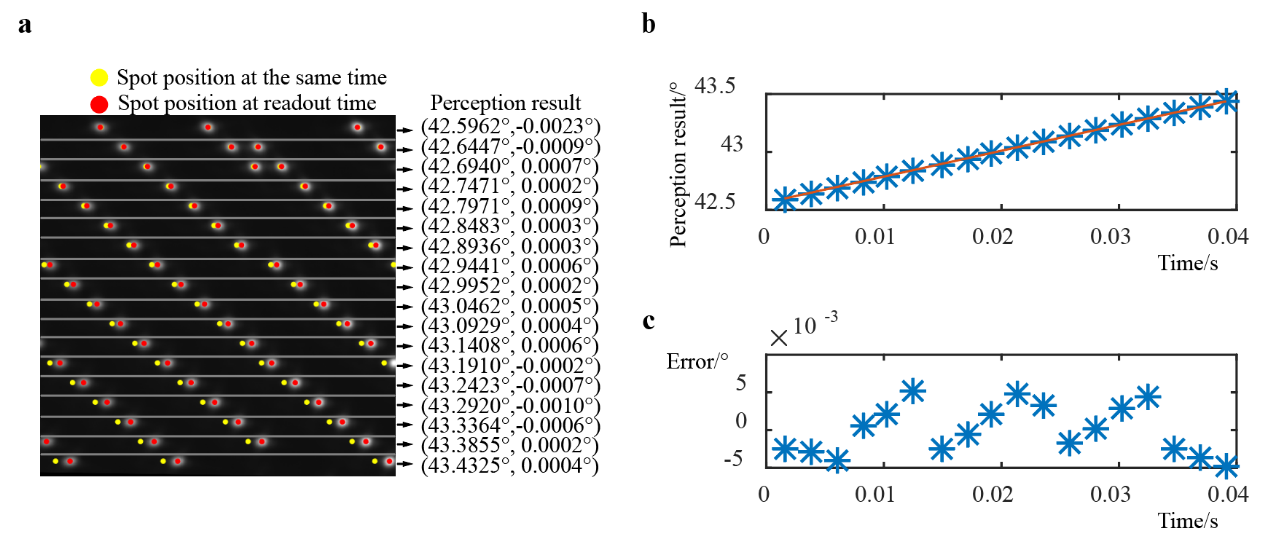


**Fig. S15** **Experiment results for high-dynamic target orientation measurement with the turntable’s rotating speed of ~22°/s.** **a** Image of the target in an arbitrary sub-FOV. **b** Orientation measurement results in the *x* direction over time. **c** Orientation measurement errors in the *x* direction over time.

**Supplementary Note 8.** Analysis of multiple sparse targets perception

LCE has the potential for multiple sparse targets perception. When multiple targets exist in the FOV of LCE, each target will form a series of light spots through the coded subeye aperture array, and these light spots are superimposed together to form the acquired image. Two perceived targets are randomly placed in the FOV, and the corresponding acquired image is shown in **Fig. S16 b**. Two correlation peaks can be formed by the correlation operation between the acquired image and the template of the coded subeye aperture array, as shown in **Fig. S16 c**. According to the positions of the two correlation peaks and the algorithm in **Supplementary Note 1**, the orientation information of the two perceived targets can be calculated.


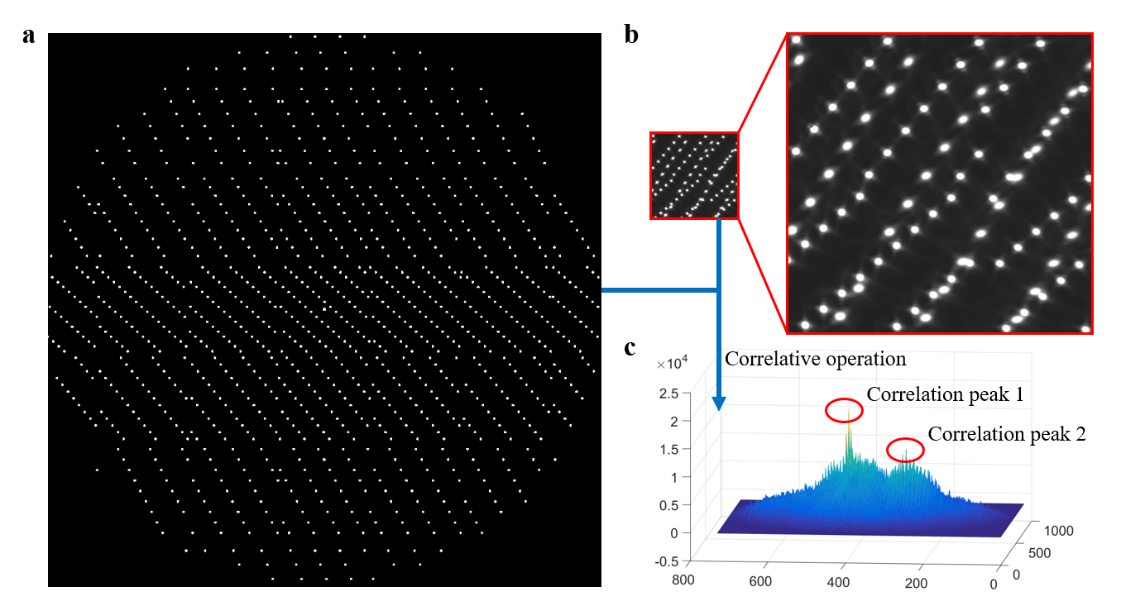


**Fig. S16** **a** Template of the coded subeye aperture array. **b** Acquired image. **c** Result of the correlation operation.

LCE has the ability of simultaneous perception for multiple sparse targets. The maximum number of perceived targets is related to the proximity of targets and the amount of identifiable coding information contained in the acquired image. As long as the correlation peaks of different targets can be identified, multiple targets can be perceived simultaneously.

**Supplementary Note 9.** Analysis of the perceived light intensity of LCE

The parameter *A* (the complex amplitude of light wave) in **Eq. (3)** is the only term reflecting the light intensity of the perceived target. The change of *A* has no influence on the profile of the diffraction spot, as shown in **Fig. S17**. By adjusting the exposure time and gain of the image sensor, the maximum intensity of the spot collected can be guaranteed to be the same or similar.


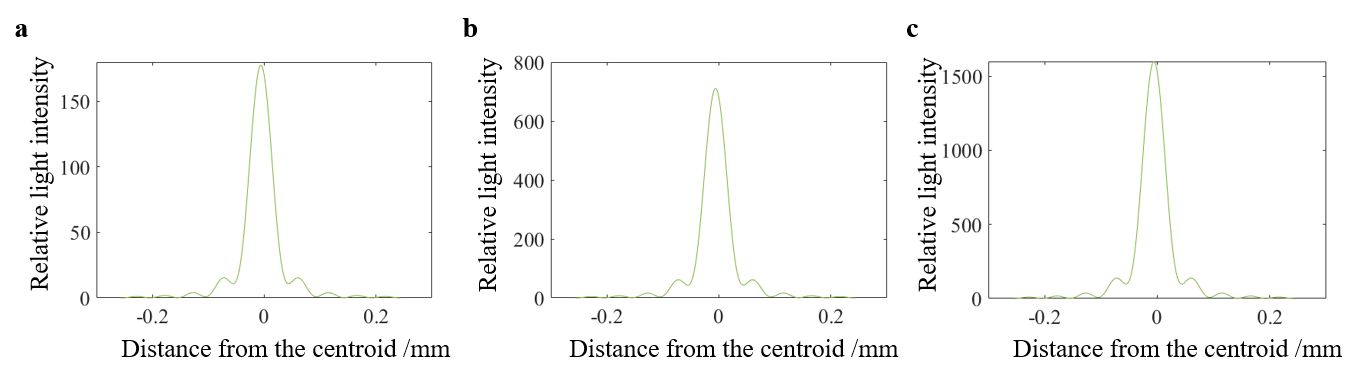


**Fig. S17** **a, b, c** are the light intensity distributions in the *x* direction of the simulated diffraction spots, when A=1, A=2 and A=3, respectively.

**Supplementary Note 10.** Diffraction analysis for parallel light

The spot profile formed by a subeye aperture is a diffraction image with its intensity distribution following the Fresnel-Kirchhoff diffraction formula [1].

$\tilde{E}\left(P\right)=\frac{A}{i\lambda}\iint_{\Sigma} \frac{\exp\left(ikl\right)}{l}\frac{\exp\left(ikr\right)}{r}\left[\frac{\cos\left(n,r\right)-\cos\left(n,l\right)}{2}\right]d\sigma$ (S8)

where *A* is a constant related to the intensity of the light source; *λ* is the wavelength of the light wave; and *k* is the wave number; $\text{d}\text{σ}$ is the integral surface element in the aperture region. See **Fig. 2b** for the meanings of vector ***n***, ***r*** and ***l***, where *r* is the norm of ***r***, *l* is the norm of ***l***.

For the aperture of a subeye, the coordinate system as shown in the **Fig. S18** is established. The center of the aperture is taken as the origin of the coordinate system. The directions of the two sides of the aperture are set as the X_0_ axis and the Y_0_ axis, and the normal direction of the aperture plane is the Z axis (the normal direction ***n*** in **Eq. (3)** points to the outside of LCE). When the light is incident at an oblique angle, the orientation vector of the light in the above coordinate system is set as (cos*α*, cos*β*, cos*γ*), and the optical path difference of a point Q (*x*_0_, *y*_0_, 0) in the aperture plane with that of the origin O is analyzed.

**Fig. S18** Diagram of optical path difference of parallel light.

Point S is the projection of the point Q on the light passing through the origin. As can be seen from **Fig. S18**, the optical path difference of the two lights is OS, and according to the Pythagorean theorem

$OS^{2}=OQ^{2}-QS^{2}$ , (S9)

Obviously

$OQ^{2}=x_{0}^{2}+y_{0}^{2}$ . (S10)

QS represents the distance from the point Q to the space vector $\vec{\text{OS}}$, which can be solved by the means of the cross product

$\begin{matrix} \overrightarrow{OQ}\times\overrightarrow{OS}= \left(x_{0},y_{0},0\right)\times\left(\cos\alpha,\cos\beta,\cos\gamma\right) \\ = \left(y_{0}\cos\gamma,-x_{0}\cos\gamma,x_{0}\cos\beta-y_{0}\cos\alpha\right) \end{matrix}$ . (S11)

The result of the cross product represents the area of the parallelogram spanning by $\vec{\text{OS}}$ and $\vec{\text{OQ}}$. Since the base of the imaginary parallelogram is a direction vector with the length of 1, the result numerically equals to the height of the parallelogram, that is, the distance between point Q and line OS. So

$QS^{2}=\left(y_{0}\cos\gamma\right)^{2}+\left(-x_{0}\cos\gamma\right)^{2}+\left(x_{0}\cos\beta-y_{0}\cos\alpha\right)^{2}$ . (S12)

And cos*α*, cos*β*, cos*γ* satisfy

$\cos\alpha^{2}+\cos\beta^{2}+\cos\gamma^{2}=1$ . (S13)

So

$\begin{matrix} OS^{2}= OQ^{2}-QS^{2} \\ = x_{0}^{2}\cos\alpha^{2}+2x_{0}y_{0}\cos\alpha\cos\beta+y_{0}^{2}\cos\beta^{2} \\ = \left(x_{0}\cos\alpha+y_{0}\cos\beta\right)^{2} \end{matrix}$ . (S14)

The optical path difference OS= *x*_0_cos*α* +*y*_0_cos*β*, that is, the optical path difference of point Q can be expressed by the angle of light and the coordinate of Q.

Combined with the above analysis, the complex amplitude of light on the subeye aperture plane can be expressed as

$\widetilde{E}_{Q}=A\exp\left[ik\left(x_{0}\cos\alpha+y_{0}\cos\beta\right)\right]$ . (S15)

The Fresnel-Kirchhoff diffraction formula becomes

$\widetilde{E}\left(P\right)=\frac{A}{\mathrm{i}\lambda}\iint_{\Sigma} \exp\left[\mathrm{i}k\left(x_{0}\cos\alpha+y_{0}\cos\beta\right)\right]\frac{\exp\left(\mathrm{i}kr\right)}{r}\left[\frac{\cos\left(\mathbf{n},\mathbf{r}\right)-\cos\left(\mathbf{n},\mathbf{l}\right)}{2}\right]d\sigma$ .(S16)

[1] *Physical Optics*. (Publishing House of Electronics Industry, 2018).
